# Non-Invasive Diagnosis for Acute Rejection Using Urinary mRNA Signature Reflecting Allograft Status in Kidney Transplantation

**DOI:** 10.3389/fimmu.2021.656632

**Published:** 2021-06-10

**Authors:** Jung-Woo Seo, Yu Ho Lee, Dong Hyun Tae, Seon Hwa Park, Ju-Young Moon, Kyung Hwan Jeong, Chan-Duck Kim, Byung Ha Chung, Jae Berm Park, Yeong Hoon Kim, Junhee Seok, Sun Hyung Joo, Seung Hwan Lee, Jong Soo Lee, Sang-Ho Lee

**Affiliations:** ^1^ Core Research Laboratory, Medical Science Institute, Kyung Hee University Hospital at Gangdong, Seoul, South Korea; ^2^ Division of Nephrology, Department of Internal Medicine, Kyung Hee University Hospital at Gangdong, Seoul, South Korea; ^3^ School of Electrical Engineering, Korea University, Seoul, South Korea; ^4^ Division of Nephrology, Department of Internal Medicine, College of Medicine, Kyung Hee University, Seoul, South Korea; ^5^ Division of Nephrology, Department of Internal Medicine, Kyungpook National University School of Medicine, Daegu, South Korea; ^6^ Division of Nephrology, Department of Internal Medicine, Seoul St. Mary’s Hospital, College of Medicine, The Catholic University of Korea, Seoul, South Korea; ^7^ Department of Surgery, Sungkyunkwan University Samsung Hospital, Seoul, South Korea; ^8^ Division of Nephrology, Department of Internal Medicine, College of Medicine, Inje University Busan Paik Hospital, Busan, South Korea; ^9^ Department of Surgery, Kyung Hee University Hospital at Gangdong, Seoul, South Korea; ^10^ Division of Nephrology, Department of Internal Medicine, University of Ulsan College of Medicine, Ulsan, South Korea

**Keywords:** kidney, transplantation, non-invasive diagnosis, acute rejection, urinary mRNA

## Abstract

Urine has been regarded as a good resource based on the assumption that urine can directly reflect the state of the allograft or ongoing injury in kidney transplantation. Previous studies, suggesting the usefulness of urinary mRNA as a biomarker of acute rejection, imply that urinary mRNA mirrors the transcriptional activity of the kidneys. We selected 14 data-driven candidate genes through a meta-analysis and measured the candidate genes using quantitative PCR without pre-amplification in the cross-sectional specimens from Korean kidney transplant patients. Expression of 9/14 genes (CXCL9, CD3ϵ, IP-10, LCK, C1QB, PSMB9, Tim-3, Foxp3, and FAM26F) was significantly different between acute rejection and stable graft function with normal pathology and long-term graft survival in 103 training samples. CXCL9 was also distinctly expressed in allografts with acute rejection in *in situ* hybridization analysis. This result, consistent with the qPCR result, implies that urinary mRNA could reflect the magnitude of allograft injury. We developed an AR prediction model with the urinary mRNAs by a binary logistic regression and the AUC of the model was 0.89 in the training set. The model was validated in 391 independent samples, and the AUC value yielded 0.84 with a fixed manner. In addition, the decision curve analysis indicated a range of reasonable threshold probabilities for biopsy. Therefore, we suggest the urine mRNA signature could be used as a non-invasive monitoring tool of acute rejection for clinical application and could help determine whether to perform a biopsy in a recipient with increased creatinine.

## Introduction

Kidney transplantation (KTx) provides better quality of life in patients with end-stage renal disease (ESRD), but KTx patients often experience allograft failure due to acute rejection (AR). Clinically applicable immune monitoring is necessary to minimize AR and prevent side effects such as infection and malignancy caused by using excess immunosuppressive drugs in kidney transplant recipients (1, 2). In the past, various tests such as ATP assay, immune cell analysis, and determination of cytokines in blood and urine have been introduced (3–7), but the usefulness of these tools for monitoring kidney graft status is yet to be evaluated in clinical trials.

Urine samples have been considered a good source of factors to monitor allograft status by biomarker researchers in the urine of KTx patients (8), because the cells contained in urine comprise various molecules that reflect the biological processes of allograft or ongoing kidney injury and because urine can be easily sampled for serial monitoring of the kidney allograft by a truly noninvasive manner in the clinical setting. In several studies previously evaluated for urine biomarkers, quantitative real-time PCR (qPCR) has been used for analyzing a few mRNAs that are biologically expected to reflect the immune status of rejection in urine. Other high-throughput approaches such as microarray analysis and RNA sequencing are ideal for the discovery of target genes associated with a specific disease within the whole transcriptome, but it is not easy to profile gene expression in urine samples because of low amounts of total RNA. Thus, we conducted a meta-analysis from public datasets of biopsy transcriptome to select proper candidates for diagnosing AR in KTx patients.

We previously established qPCR assays to measure absolute and relative amounts of urinary mRNAs using 18S rRNA as a reference (9). In this study, we identified biomarkers for diagnosing AR in urinary cells using the qPCR for absolute quantification without pre-amplification. We developed a signature to distinguish patients with AR from patients with stable graft function (STA). After validation in independent samples (n=391), and by using decision curve analysis, we evaluated whether the signature is useful for physicians in deciding to perform kidney allograft biopsy.

## Materials and Methods

### Patients

The Assessment of immunologic Risk and Tolerance in Kidney Transplantation (ARTKT-1) study was a cross-sectional sample collection study, which enrolled 543 KTx patients at Kyung Hee University Hospital at Gangdong from 2012 to 2015, and five different hospitals (Kyung Hee Medical Center, Kyungpook National University Hospital, Samsung Medical Center, Seoul St. Mary’s Hospital of the Catholic University of Korea, and Inje University Busan Paik Hospital) from 2013 to 2015. We used the Modification of Diet in Renal Disease (MDRD) equation to estimate the glomerular filtration rate (GFR). Indication biopsy was taken upon any clinical indication, but protocol biopsy was performed at 2 weeks (KHG and KMC) or 3 months (CMC) based on the institution’s policy, and 457 biopsy-proven samples classified by pathologists at each hospital who were unaware of the analyzed results of the samples using their clinical status and Banff 2007 classification (10) and 86 non-biopsied patients were enrolled.

In 457 biopsy-proven samples, there were normal pathology (NP, n=119), acute T cell-mediated rejection (TCMR, n=75), borderline changes (BC, n=58), acute antibody-mediated rejection (ABMR, n=26), chronic active antibody-mediated rejection (cABMR, n=25), BK virus nephropathy (BKVN, n=15), acute tubular necrosis (ATN, n=30), calcineurin inhibitor toxicity (CNI, n=28), glomerulonephritis (GN, n=27), interstitial fibrosis/tubular atrophy (IF/TA, n=12), and others (n=42). In addition, we enrolled 79 non-biopsied patients defined as long-term graft survival (LTGS) with maintaining stable kidney function while on maintenance immunosuppression more than 10 years after KT and 7 non-biopsied patients defined as operational tolerance (OT) with maintaining stable kidney function (serum creatinine < 1.5 mg/dl) without any immunosuppressive drugs for more than 1 year. We excluded 42 biopsy-proven samples with acute pyelonephritis, acute TI nephritis, diabetic nephropathy, hydronephrosis, hypertensive glomerulopathy, microvascular injury, non-specific injury, unknown, and 7 OT samples because of small scale, less frequent findings, or unclearly defined specimens for pathological classification in this study.

At the time of transplantation, none of the transplant donors were from a vulnerable population and all donors or next of kin provided written informed consent that was freely given. All kidney transplant patients provided written informed consent to participate in the study, and the study was approved by the local institutional review board at Kyung Hee University Hospital at Gangdong (#2012-030, KHNMC) and registered with the Clinical Research Information Service (KCT0001010).

### Selection for AR Candidate Genes

To select AR candidate genes, we searched the keyword “kidney rejection” in the GEO database. Among the initially queried results, we filtered further by species (Homo Sapiens) and data sets using the gene expression platform (Affymetrix GeneChip U133+2) to minimize the unexpected bias by platform difference. We manually examined the top 10 data sets in terms of the sample sizes and excluded sets that did not have acute rejection and stable samples. We found the significantly different genes between STA and AR using the GeneMeta R package in four data sets (**Supplemental Table 1**), following the approach of Choi et al. (11). In total, 137 probe-sets corresponding to 109 unique genes were found to be significant across multiple data sets (FDR<0.01 and FC>2) by false discovery rates (FDRs) obtained from 1,000 permutations. The effective fold change of the meta-analysis was calculated as the average fold change of the four data sets weighted by the number of samples. Among them, we selected the top 10 genes in the order of fold changes with the clarity of gene annotation excluding multiple probes mapped to a gene or a probe mapped to multiple genes and with the easiness of PCR primer design (**Supplemental Table 2**).

In addition, we selected representative genes (CD3ϵ, Foxp3, OX40, and Tim-3) that are well known as diagnostic markers for allograft rejection through literature review (**Supplemental Table 3**).

### Urine Collection and qPCR by Absolute Quantitation

Urine (about 50 ml) in each center were collected at the time of biopsy (for biopsied patients) or visiting the hospital (for patients with long-term survival graft). We immediately centrifuged and stored samples in each center’s laboratory after collection. All centers followed an identical protocol with urine collection at the clinical sites for the sedimentation of urinary cells. After the urine was centrifuged at 2,000x g for 20 min, the pellet was transferred into RNAlater and stored at -80°C until use.

Total RNA was extracted from the urinary pellets using a PureLink RNA Mini Kit (Invitrogen) according to the manufacturer’s recommendations. The quantity (absorbance at 260) and purity (the absorbance at 260/280) of RNA were measured using a NanoDrop^®^ ND-2000 UV spectrophotometer (Thermo Scientific). The median (25^th^ and 75^th^ percentile) quantity (µg) of total RNA was 0.74 (0.250-1.680), and the median (25^th^ and 75^th^ percentile) purity of RNA was 1.85 (0.50-2.01) in 494 total samples. Also, we assessed the quality of urinary RNA using 18S rRNA ≥ 1x10^4^ copies/ug and TGF-β1 ≥ 1x10^2^ copies/ug as quality control (QC) parameters for the improvement of data quality before the measurement of urinary mRNA levels. In 494 total samples, 402 samples (81%) passed QC (**Supplemental Table 4**).

Reverse transcription was performed with total RNA using M-MLV RT enzyme (200 U/µl; Mbiotech, Inc., Seoul, Korea), and absolute quantities of the mRNAs were measured by TaqMan probe qPCR assays without pre-amplification step. Each DNA oligo serially diluted from 1x10^-1^ to 1x10^-8^ ng/ul was used for a standard curve. The copy number of each mRNA was calculated using the molecular weight of DNA and the standard curve. The undetected value of mRNA was calculated by replacing 40 C_t_ value in the blank for data analysis. The mRNA values were then normalized by 18S rRNA copies (x10^-6^) used as endogenous control and log_10_-transformed to reduce deviation before being used in data analysis (9). 

### *In Situ* Hybridization Assays

We performed *in situ* hybridization (ISH) assay for CXCL9 expression in renal biopsy tissues (3 NP, 3 acute TCMR, and 3 acute ABMR) using the RNAscope 2.5 assay kit (Advanced Cell Diagnostics, Hayward, CA, USA) according to the manufacturer’s recommendations. Briefly, formalin-fixed paraffin embedded sections were subjected to deparaffinization, proteolytic digestion with enzyme denaturation, and hybridization with a CXCL9 probe. The RNAscope target was retrieved at 95°C for 15 min and then incubated with RNAscope enzyme for 15 min. The hybridization of the probes for CXCL9, human peptidylprolyl isomerase B (positive control), and diaminopimelate B (negative control) were incubated at 40°C for 2 hours. The preamplifier, signal enhancer, amplifier, and label probe were sequentially incubated with samples at 40°C for 30, 15, 30, and 15 min, respectively. After each step, sections were washed two times, and hybridization signals were detected by 3,3-diaminobenzidine (DAB) staining, followed by counterstaining with Gill’s hematoxylin.

### Statistical Analysis

We compared the levels of transcripts between the STA and AR groups by the non-parametric Mann-Whitney test and assessed differences among the STA, AR, BKVN, and OGIs groups by the non-parametric Kruskal-Wallis test using SPSS for Windows, version 20.0 (IBM Corp, Armonk, NY).

We developed a model to predict AR by a binary logistic regression with all significantly altered genes (12). To diagnose AR using a model, we used a decision cutoff point maximizing Youden’s index, which is the sum of sensitivity and specificity (13). Receiver operating characteristic (ROC) curve analyses and decision curve analyses were conducted to evaluate the model (14, 15).

## Results

### Study Design and Clinical Characteristics of Patients

**Figure 1:**
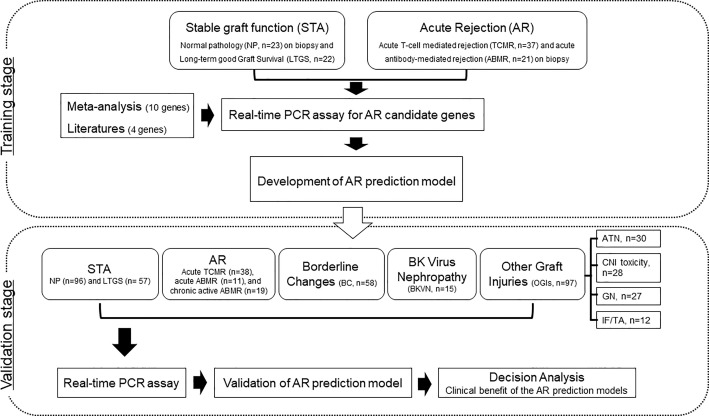
Workflow of biomarker discovery and validation for the diagnosis of AR in urine pellets. After a meta-analysis and review of the published literature to obtain candidate genes for diagnosis of AR, we measured the levels of 103 training samples composed of stable (STA, n=45), and acute rejection (AR, n=58) using absolute qPCR methods. We developed a signature by combining significantly different transcripts to distinguish AR from STA. We validated significant mRNAs and tested the performance of the diagnostic signature in 391 independent samples including STA (n=153) composed of NP (n=96) and long-term graft survival (LTGS, n=57), AR (n=68) composed of acute TCMR (n=38), acute ABMR (n=11), and chronic active ABMR (n=19), borderline changes (BC, n=58), BKVN (n=15), and other graft injuries (OGIs, n=97) including acute tubular necrosis (ATN, n=30), calcineurin inhibitor (CNI, n=28) toxicity, glomerulonephritis (GN, n=27), and interstitial fibrosis/tubular atrophy (IF/TA, n=12). We also performed a decision curve analysis to determine the value of the signature in predicting AR.

We supplemented LTGS samples for a small number of normal pathology group as STA and split into a training set of AR (n=58) and STA (n=45) groups randomly selected by a clearly defined pathology. We first measured 14 genes in 103 training samples for gene selection by absolute qPCR method without pre-amplification, and then developed an AR model by statistical analysis. In the next step, we measured 6 genes to verify the discriminative power of the model in 391 independent samples, which consist of STA (n=153), AR (n=68), borderline changes (BC, n=58) for TCMR, BKVN (n=15), and other graft injuries (OGIs, n=97) including acute tubular necrosis (ATN, n=30), calcineurin inhibitor (CNI, n=28) toxicity, glomerulonephritis (GN, n=27), and interstitial fibrosis/tubular atrophy (IF/TA, n=12). We assessed the AR prediction model with the fixed cutoff point and evaluated whether the decision curve analysis of the signature is better than biopsy for clinical management and diagnosis of AR. The work-flow chart of this study is shown in **Figure 1**.

There was no significant difference in the mean age of patients and maintenance immunosuppression between the groups. The duration after KT was significantly longer in the STA group than others because this group contained the patients with long-term good graft function, whose follow-up periods after KT were over 10 years. STA group showed a lower proportion of deceased donor KT and low HLA mismatching numbers than other groups. Residual graft function was significantly high in the STA group compared to others in both the training and validation set. The baseline clinical characteristics of the study population are summarized in **Table 1**.

**Table 1:** Baseline characteristics and clinical parameters of enrolled patients by study group.

	Training set (n=103)			Validation set (n=391)					
	STA^A^	AR^B^	*p* value^†^	STA^C^	AR^D^	Borderline changes	BKVN	OGIs^E^	*p* value^‡^
Number of patients (n)	45	58	–	153	68	58	15	97	–
Age (years)	49.7 ± 11.5	47.2 ± 10.9	0.263	48.7 ± 12.0	46.7 ± 10.6	48.3 ± 11.5	47.7 ± 15.7	47.7 ± 11.8	0.823
Sex (Male, %)	17 (37.8)	35 (60.3)	0.023	98 (64.1)	47 (69.1)	45 (77.6)	11 (73.3)	65 (67.0)	0.430
Duration after KT (Months)	92.2 ± 102.0	28.8 ± 43.7	<0.001	73.6 ± 98.5	40.2 ± 48.8	7.9 ± 15.1	11.7 ± 10.5	46.5 ± 64.4	<0.001
Deceased donor KT (n, %)	14 (31.1)	23 (39.7)	0.370	47 (30.7)	31 (45.6)	42 (43.3)	5 (33.3)	42 (43.3)	0.009
ABO incompatible KT (n, %)	4 (8.9)	13 (22.4)	0.068	21 (13.7)	10 (14.7)	6 (10.3)	2 (13.3)	3 (3.1)	0.092
HLA mismatching (n)	3.0 ± 1.9	3.5 ± 1.5	0.127	3.1 ± 1.5	3.7 ± 1.8	3.7 ± 1.4	3.8 ± 1.7	3.2 ± 1.4	0.012
Induction immunosuppression									
Basiliximab (n, %)	34 (75.6)	39 (67.2)	0.357	105 (68.6)	52 (76.5)	38 (65.5)	10 (66.7)	73 (75.3)	0.466
Anti-thymocyte globulin (n, %)	11 (24.4)	19 (32.8)		48 (31.4)	16 (23.5)	20 (34.5)	5 (33.3)	24 (24.7)	
Maintenance immunosuppression									
Steroid (n, %)	32 (71.1)	55 (94.8)	0.001	135 (88.2)	56 (82.4)	52 (90.7)	14 (93.3)	88 (90.7)	0.504
Tacrolimus (n, %)	29 (64.4)	38 (65.5)	0.910	122 (79.7)	50 (73.5)	54 (93.1)	14 (93.3)0	80 (82.5)	0.042
Cyclosporine (n, %)	13 (26.7)	17 (29.3)	0.767	26 (17.0)	11 (16.2)	3 (5.2)	0 (0)	13 (13.4)	0.031
Mycophenolate mofetil (n, %)	32 (71.1)	48 (82.8)	0.159	134 (87.6)	50 (73.5)	52 (89.7)	10 (66.7)	82 (84.5)	0.020
mTOR inhibitor (n, %)	5 (11.1)	3 (5.2)	0.266	5 (3.3)	8 (11.8)	3 (5.2)	1 (6.7)	3 (3.1)	0.619
eGFR (ml/min/1.73m^2^)	77.8 ± 17.7	36.8 ± 21.0	<0.001	72.3 ± 20.8	36.0 ± 18.5	53.0 ± 18.5	32.1 ± 10.5	40.7 ± 22.3	<0.001
Donor information									
Age (years)	39.9 ± 13.6	48.5 ± 11.8	0.001	43.3 ± 13.8	44.7 ± 14.2	49.8 ± 12.0	54.1 ± 8.3	44.3 ± 13.4	<0.001
Sex (Male, %)	28 (62.2)	34 (58.6)	0.711	76 (49.7)	35 (51.5)	33 (56.9)	6 (40.0)	48 (49.5)	0.709

Data are expressed as mean ± standard deviation or number (percentage).

STA, stable allograft function; AR, acute rejection; BKVN, BK virus associated nephropathy; OGIs, other graft injuries; KT, kidney transplantation; HLA, human leukocyte antigen; eGFR, estimated glomerular filtration rate; mTOR, mammalian target of rapamycin

^A^STA group in the discovery set consisted of normal pathology (n=23) and long-term graft survival (n=22).

^B^AR group in discovery set consisted of acute T cell-mediated rejection (n=37), and acute antibody-mediated rejection (n=21).

^C^STA group in the validation set consisted of normal pathology (n=96) and long-term graft survival (n=57).

^D^AR group in validation set consisted of acute T cell-mediated rejection (n=38), acute antibody-mediated rejection (n=11), and chronic active antibody-mediated rejection (n=19).

^E^OGIs include acute tubular necrosis (n=30), calcineurin inhibitor toxicity (n=28), glomerulonephritis (n=27), and interstitial fibrosis/tubular atrophy (n=12).

^†^For non-normally distributed variables, data were analyzed using the non-parametric Mann-Whitney test.

^‡^ For non-normally distributed variables, data were analyzed using the Kruskal-Wallis test.

### Urinary mRNA Levels in the Training Set and ISH in Biopsy Tissues

**Figure 2:**
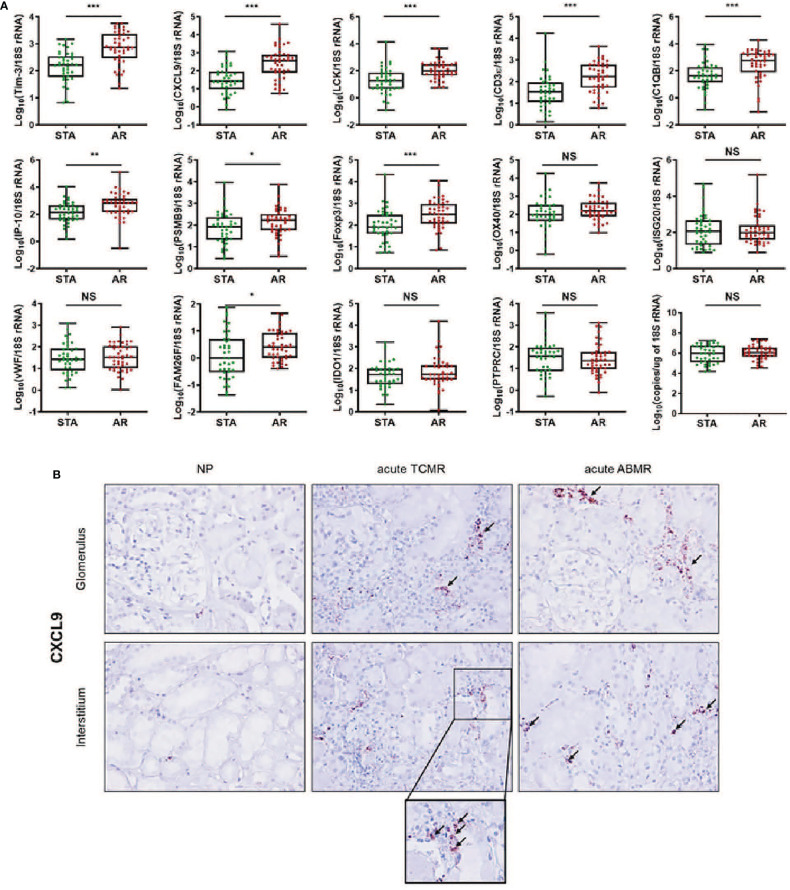
The expression levels of each mRNA between STA (n=45) and AR (n=58) were analyzed using absolute quantitative qPCR without pre-amplification. Each mRNA level was log10-transformed after each mRNA copy number was normalized with 18S rRNA copies (x10-6) in the QC-passed samples (STA, n=40; AR, n=44). **(A)** The levels of CXCL9, IP-10, C1QB, PSMB9, LCK, CD3e, Foxp3, FAM26F, and Tim-3 mRNAs were significantly elevated in AR compared to STA, and for OX40, ISG20, vWF, IDO1, and PTPRC mRNAs, there was no difference. In the 18s rRNA used as an endogenous control, there was no difference between AR and STA. P values by the non-parametric Mann-Whitney test were expressed as the mean ± SE. NS: not significant, *P < 0.05, **P < 0.01 and ***P < 0.001 versus STA. Although LCK, Foxp3, and FAM26F mRNAs were statistically significant, these mRNAs were not detected in more than 10% of the QC-passed samples. Therefore, we excluded these mRNAs for further analysis. **(B)** CXCL9 mRNA expression in kidney biopsy tissues of NP, acute TCMR and acute ABMR groups was examined by ISH (original magnification x400). CXCL9 was distinctly expressed in the damaged tubules in kidney allografts of acute TCMR and predominantly in the peritubular capillary area in ABMR groups (black arrows). Scale bars: 50 μm.

We measured the expression levels of 14 genes using absolute qPCR without the pre-amplification step in the training set. If mRNA is undetected, the absolute value of the mRNA was calculated with the standard curve by replacing 40 C_t_ value in the blank with undetected value, and each mRNA level was log_10_-transformed after normalization with 18S rRNA copies (x10^-6^). The levels of CXCL9, C1QB, LCK, CD3ϵ, Foxp3, Tim-3 (P<0.001 for each mRNA), IP-10 (P<0.01), PSMB9, and FAM26F (P<0.05) were significantly elevated in the AR group compared to STA, but for OX40, IDO1, ISG20, vWF, and PTPRC there was no difference (**Figure 2A** and **Supplemental Table 5**). The copy number of 18S rRNA per total RNA amount showed no difference between the AR and STA groups. Although LCK, Foxp3, and FAM26F mRNAs were statistically significant, these mRNAs were not detected in 14% (n=12), 26% (n=22), and 58% (n=49) of the QC-passed samples (n=84), respectively. Thus, we excluded these mRNAs (FAM26F, Foxp3, and LCK) due to low detectable frequency and (OX40, IDO1, ISG20, vWF, and PTPRC) with no statistical difference between AR and STA for further analysis. In addition, we investigated representatively CXCL9 level using *in situ hybridization* (IHS) analysis to confirm whether the mRNA level in kidney biopsy tissue is consistent with the result of urinary mRNA by qPCR assay. CXCL9 was distinctly expressed in the damaged tubules in the kidney allografts of acute TCMR and predominantly in the peritubular capillary area in ABMR groups, consistent with the results of qPCR analysis (**Figure 2B**).

### Development of Prediction Signature to Distinguish AR From STA

We performed ROC curve analysis to evaluate each target for AR diagnosis, and the AUC values of the individual gene were not sufficient to distinguish AR from STA (**Supplemental Table 6**). Therefore, to improve the diagnostic accuracy of AR, we developed an AR prediction model using a binary logistic regression in the training set.

**Figure 3:**
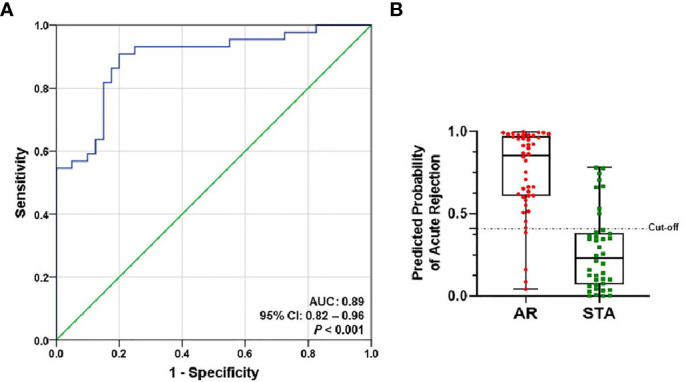
ROC curve analysis of the signature to distinguish AR from STA in the training set. The graphs of receiver operating characteristic (ROC) curves and the predicted probability of AR for the six-genes signature show discrimination of AR from STA. **(A)** The area under the curve (AUC) value of the six-gene model is 0.89 (95% CI, 0.82-0.96; P<0.001). **(B)** In the graph of the predicted probability of AR with the cutoff point (0.40889), the signature yielded 86% accuracy, 91% sensitivity, 80% specificity, 83% positive predictive value (PPV), and 89% negative predictive value (NPV).

The level of each mRNA was log_10_-transformed after each mRNA copy number was normalized with 18S rRNA (x10^-6^). The AUC value of the signature was 0.89 (95% CI, 0.82-0.96; P<0.001) (**Figure 3A**). The equation of a six-gene signature is shown below:


$$ln\frac{p(x)}{1-p(x)}=-6.582+(0.404\;\times \;C1QB)+(0.998\;\times \;CD3\epsilon )+(2.206\;\times \;CXCL9)+(-0.904\;\times \;IP10)+(-1.829\;\times \;PSMB9)+(2.207\;\times \;Tim3)$$

With the cutoff point (0.40889), the predicted probability of the signature also yielded 86% accuracy, 91% sensitivity, 80% specificity, 83% positive predictive value (PPV), and 89% negative predictive value (NPV) (**Figure 3B** and **Table 2**). The signature improved the diagnosis of AR from STA compared to Tim-3 alone.

**Table 2:** Prediction performance of the signature between AR and STA in the training set.

Distinction	AUC (95% CI)	Sensitivity (95% CI)	Specificity (95% CI)	PPV (95% CI)	NPV (95% CI)	Accuracy (95% CI)	Disease prevalence (95% CI)	Cut-off point	P value
AR *vs* STA	0.89 (0.824 - 0.962)	90.91 (78.33% - 97.47%)	80.00 (64.35% - 90.95%)	83.33 (72.76% - 90.35%)	88.89 (75.63% - 95.38%)	85.71 (76.38% - 92.39%)	52.38 (41.19% - 63.40%)	0.408887	<0.0001

PPV, positive predictive value; NPV, negative predictive value.

### Validation of the Diagnostic Signature to Predict AR

We measured six genes in 391 independent samples including STA, AR, BC, BKVN, and OGIs groups, and evaluated the discriminative power of the diagnostic signature. All of the 6 genes were significantly increased in the BKVN group as well as the AR group compared to STA by Kruskal-Wallis nonparametric test and Dunn’s post-hoc test for multiple comparisons. Interestingly, the expression of CXCL9 and IP-10 genes (P<0.01) in BKVN was significantly higher rather than those in the AR group. Compared to STA group, 4 genes (Tim-3, P < 0.05; CXCL9, P < 0.01; CD3ϵ, P < 0.001; C1QB, P < 0.05) in the BC group and 5 genes (Tim-3, P < 0.001; CXCL9, P < 0.01; CD3ϵ, P < 0.05; C1QB, P < 0.001; PSMB9, P < 0.001) in the OGIs group were significantly up-expressed. In addition, the expression levels of all six genes in the AR group were significantly higher than those in the BC and OGIs groups. Scatter plots of the mRNAs and the comparisons between groups by Mann-Whitney t test are shown in **Supplemental Table 7** and **Supplemental Figure 1**.

**Figure 4:**
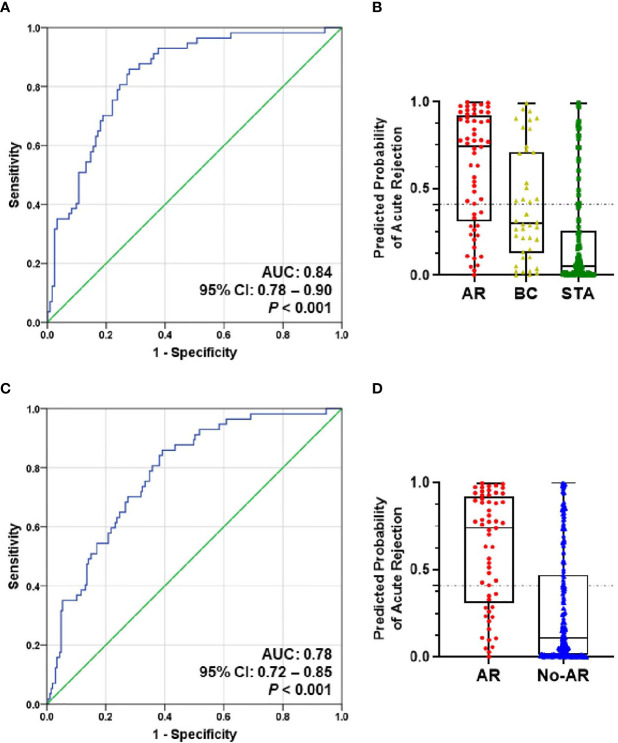
The prediction performance of the signature in the validation set. **(A)** ROC curve of the AR predicted probability for distinguishing AR (n=57) from STA (n=122) in the QC-passed samples shows the AUC of 0.84 (95% CI, 0.78-0.90; P<0.001). **(B)** The box plot shows the AR predicted probability of the signature with the fixed cut-off point (0.40889) in AR (n=57), BC (n=42), and STA (n=122), and the signature yielded 77% accuracy, 70% sensitivity, 80% specificity, 63% positive predictive value (PPV), and 85% negative predictive value (NPV). The horizontal line within each box represents the median, the bottom and top of each box represents the 25th and 75th percentile value, and the I bar represents the 5th and 95th percentile value. The plus symbol represents the mean, and the dots indicate outliers. **(C)** ROC curve of the predicted probability for distinguishing AR from No-AR (STA + OGIs, n=207) in the QC-passed samples is shown. The AUC value of the signature was 0.78 (95% CI, 0.72-0.85; P<0.001). **(D)** The box plot shows the AR predicted probability of the signature with the fixed cut-off point (0.40889) in AR (n=57) and No-AR (n=207), and the signature yielded 72% accuracy, 70% sensitivity, 72% specificity, 41% positive predictive value (PPV), and 90% negative predictive value (NPV).

To evaluate the performance of the AR predicted signature with the fixed cut-off point, we tested its performance in AR (n=57) and STA (n=122) in the QC-passed samples. BC group was not included in the AR group because there are significant differences in the six genes between the AR and BC groups. The AUC value of the signature yielded 0.84 (95% CI, 0.78-0.90; P<0.001) (**Figure 4A**), and the predicted probability of the signature showed well discrimination of AR from STA, but it was difficult to discriminate BC (n=42) from AR or STA in the validation set (**Figure 4B**). With the fixed cutoff point, the signature displayed 70% sensitivity (95% CI, 57%-82%) and 80% specificity (95% CI, 72%-87%). The overall performance of the signature yielded 77% accuracy, 63% PPV, and 85% NPV (**Table 3**). Furthermore, we assessed the potential of the signature for distinguishing the AR from the no-AR group (STA and OGIs, n=207) in the QC-passed samples excluding the BC and BKVN groups. The signature yielded an AUC value of 0.78 (95% CI, 0.72-0.85; P<0.001) with 70% sensitivity (95% CI, 57%-82%), 72% specificity (95% CI, 65%-78%), 77% accuracy, 63% PPV, and 85% NPV (**Figures 4C,D** and **Table 3**). In distinguishing the AR from OGIs group (OGIs, n=85), the signature yielded an AUC value of 0.70 (95% CI, 0.62-0.79; P<0.001) with 70% sensitivity (95% CI, 57%-82%), 60% specificity (95% CI, 49%-71%), 64% accuracy, 54% PPV, and 75% NPV (**Table 3**).

**Table 3:** Prediction performance of the signature in the validation set by the fixed cut-off point.

Distinction	AUC (95% CI)	Sensitivity (95% CI)	Specificity (95% CI)	PPV (95% CI)	NPV (95% CI)	Accuracy (95% CI)	Disease prevalence (95% CI)	P value
AR *vs* STA (n = 179)	0.84 (0.778 - 0.899)	70.18 (56.60% - 81.57%)	80.33 (72.16% - 86.97%)	62.5 (52.85% - 71.25%)	85.22 (79.31% – 89.66%)	77 (70.24% - 83.03%)	31.84 (25.09% - 39.21%)	<0.0001
AR *vs* OGIs (n=142)	0.703 (0.617 - 0.789)	70.18 (95% CI: 56.60 - 81.57)	60.00 (95% CI: 48.80 - 70.48)	54.05 (95% CI: 46.31 - 61.61)	75.00 (95% CI: 66.02 - 82.24)	64 (95% CI: 55.61 - 71.96)	40.14 (32.01 to 48.69)	<0.0001
AR *vs* STA + OGIs (n = 264)	0.783^*^ (0.721 - 0.845)	70.18 (56.60% - 81.57%)	71.98 (65.33% - 77.98%)	40.82 (34.35% - 47.62%)	89.76 (85.37% - 92.94%)	72 (65.74% - 76.95%)	21.59 (16.78% - 27.05%)	<0.0001

*AUC was calculated based on AR predicted probability of the signature for patients including AR (n=57), STA (n=122), OGIs (n=85), and no-AR (STA and OGIs, n=207) in QC-passed samples. PPV, positive predictive value; NPV, negative predictive value.

### Decision Curve Analysis of the Diagnostic Signature

**Figure 5:**
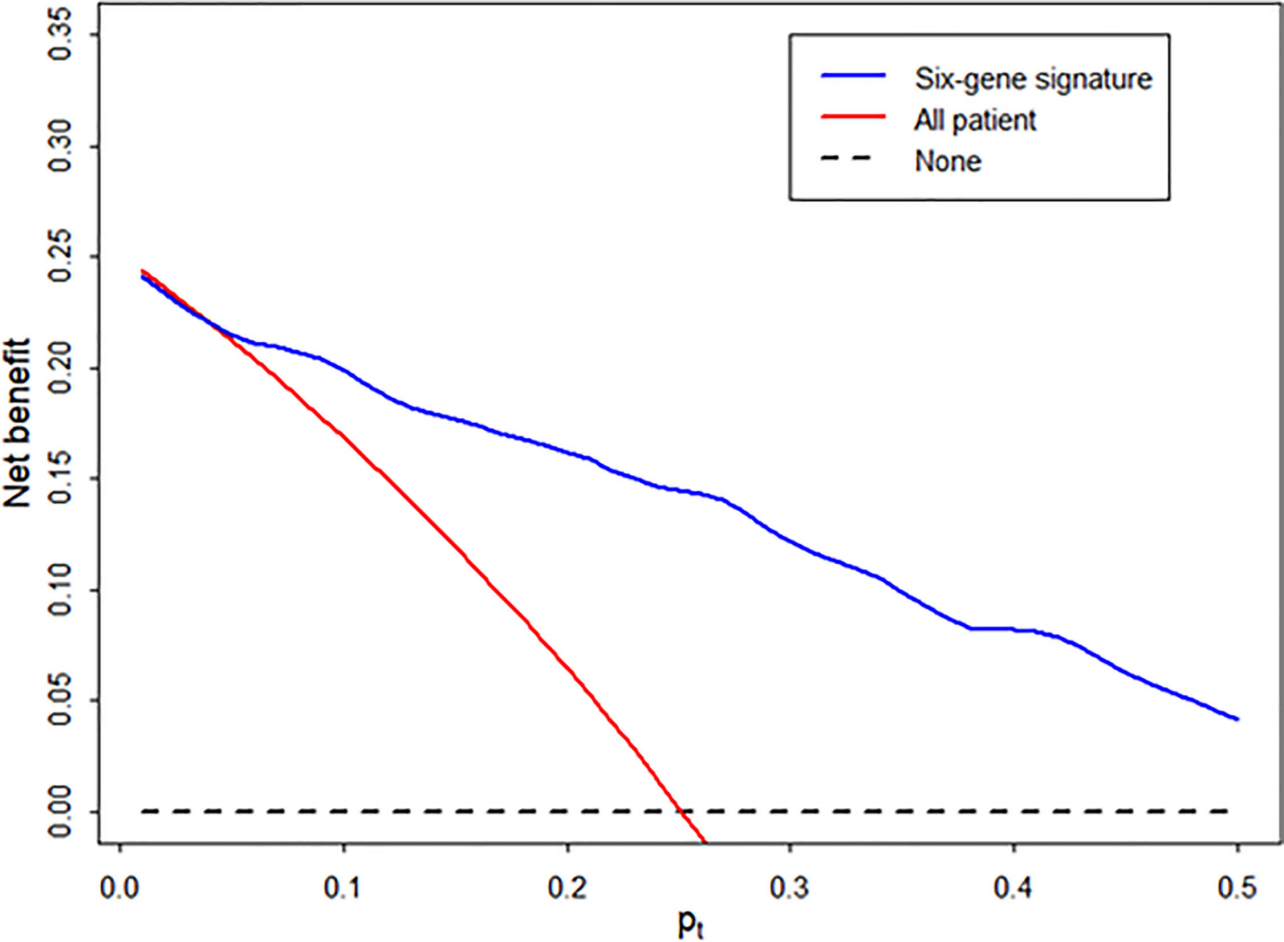
Decision curve to evaluate the clinical benefit of the signature to distinguish AR from STA. We performed decision curve analysis to assess the clinical benefit of the diagnostic signature using the predicted probability for each patient in independent samples and determined whether the signature can help avoid the number of unnecessary biopsies for the diagnosis of AR. In the decision curve, the y-axis represents the net benefit ((true-positive count/n)-(false-positive count/n)x[*p_t_*/(1-*p_t_*)]), where the true-positive count is the number of patients with AR, the false-positive count is the number of patients with STA, n is the total number of patients, and *p_t_* is the threshold probability. Here, *p_t_*/(1- pt) is the ratio of the harm caused by a false positive compared to that caused by a false negative. The blue line represents the net benefit of the diagnostic signature. The red line represents the net benefit of the biopsy strategy in all patients. The black line, which represents no net benefit, is the strategy without biopsy.

Using decision curve analysis, we assessed whether the analysis of the signature was better than biopsy for the clinical management and diagnosis of AR (**Figure 5**). Based on the decision curve analysis, the range of reasonable threshold probabilities (*p_t_*) with the highest net benefit was from 0.2 to 0.5 for the diagnostic signature. Ultimately, the diagnostic signature within a reasonable threshold probability may complement or justify allograft biopsy for diagnosing AR after kidney transplantation.

## Discussion

The expression of AR-specific mRNAs in urinary cell pellets for monitoring the immune status of kidney transplant patients has been studied by many investigators in the last decade. Absolute quantification with preamplification, which was developed by Suthanthiran et al., has been regarded as the standard method for biomarker research using urinary mRNA quantification in kidney transplantation (16). Although preamplification is reported to be effective in the detection of a very low amount of mRNAs, there are several concerns regarding the clinical application of preamplification assisted qPCR, such as inconsistent preamplification efficiency, reproducibility, and specificity (17–19). In this study, we conducted qPCR in the absence of the preamplification process and showed that more than 80% of target mRNAs (11/14) were stably measured in most patients of the training set. Although LCK, Foxp3, and FAM26F genes were statistically significant, the undetected rate of LCK, Foxp3, FAM26F, and vWF genes of 14 candidate genes was 14%, 26%, 58%, and 60% in the training set, respectively. However, for the subsequent analysis, we did not strictly use the genes with over 10% undetected value in the training set. Some genes with large undetermined data may be due to the reason without preamplification. Foxp3 mRNA has been suggested as a noninvasive biomarker for AR, but a study recently reported that Foxp3 mRNA was undetectable in most patients with acute tubular injury (20). We also observed similar results in most of the samples and thus did not include Foxp3 mRNA in data analysis (21, 22). Even so, we suggest that qPCR assay without preamplification be an available method for the measurement of AR-specific urinary mRNAs.

We performed a meta-analysis with GEO data sets on biopsy samples from patients with AR and STA to search reasonable molecules and identified 10 genes. Moreover, in gene set enrichment analysis, most of the pathways in total were related to the immune signaling pathway, and 9 genes among the 10 candidates identified by meta-analysis were associated with inflammatory response and the immune system (**Supplemental Figure 2**). We also selected an additional 4 genes (CD3ϵ, Foxp3, OX40, and Tim-3), because urinary mRNA studies suggested that the expression level of CD3ϵ, Foxp3, OX40 or Tim-3 mRNA was higher in patients with AR than in patients with STA, and thus the measurement of these mRNAs may be a promising noninvasive tool for the diagnosis and prediction of AR (16, 23–25). Additionally, in our ISH study, CXCL9 was distinctly expressed in the damaged tubules in kidney allografts of acute TCMR and predominantly in the peritubular capillary area in ABMR groups, consistent with the results of qPCR analysis, but not in those of the STA group. Thus, we showed that the result using ISH analysis was identical to the result obtained by qPCR analysis and that cells in urine could well reflect ongoing kidney injuries or kidney allograft status. Furthermore, five genes of our six-gene set were matched to 5 of the 11 genes used in the uCRM study. Sigdel’s study analyzed the public transcriptome expression datasets from AR or STA Kidney, lung, heart, and liver transplanted biopsies. The total number of patients used in their study was 236 samples, of which only 52 were from kidney transplants (8). Their study suggested 11 genes with equally increased expression in graft rejection, regardless of organ type. Since the mechanism of graft rejection would not differ greatly depending on the organ type, it is natural that some of our genes overlapped to a part of the uCRM genes. However, our study applied four public datasets for meta-analysis, including subsequent studies on tissue transcriptome from kidney transplant patients, and analyzed a total of 654 kidney transplant patients. Therefore, we presumed that our candidate gene set might be more useful to predict kidney rejection.

In most previous studies investigating the novel biomarkers for rejection, molecular signatures were not locked down while they were examined in the validation set (26). In this study, the prediction model using our diagnostic signature was importantly fixed without any further modification and showed good discrimination potential with an AUC of 0.84 in independent validation, suggesting that it is highly likely to be reproducible in clinical practice. In most of the previous biomarker studies in the field of solid organ transplantation, the model generated from the training group was rarely applied to the validation group as a fixed model. We verified the usefulness of this fixed gene signature model in the validation set of the patients in the ARTKT study, which prospectively collected urine samples at the time of allograft biopsy. However, the signature was limited in distinguishing AR from BC and BKVN because all six genes were also significantly elevated in the BC and BKVN group compared to STA, like rejection. To confirm the performance of the discrimination from not only the STA group but also other graft injuries, the six-genes signature was evaluated for discrimination from OGIs including ATN, CNI, GN, and IF/TA injuries. The discriminant power of the signature to distinguish from OGI was slightly lower than that of the STA group. Recurrent or *de novo* glomerulonephritis was about 30% in the OGIs group, and we think that the ability to distinguish AR from OGIs was slightly lower than that of the STA group because glomerulonephritis is primarily mediated by an immunological injury in the pathophysiology. Furthermore, in decision curve analysis to assess the performance of the signature for clinical benefit, the diagnostic signature had the highest net benefit than biopsy at a *p_t_* of 0.2. Therefore, our signature is low in terms of relative harm and costs for patients compared to kidney biopsy.

The BK virus is a major causative agent of nephropathy and can lead to the deterioration of the transplanted kidney and graft failure (27). Several biomarkers have been proposed for the diagnosis of BKVN, such as heat shock protein 90-α (28), CXCL9 (29), neutrophil gelatinase-associated lipocalin (30), and BK virus-specific CD4 T cells (31); however, none of the biomarkers have demonstrated superiority over viral DNA test in blood and urine. These results are explained by the fact that a substantial proportion of patients with BKVN also have AR or that the mechanism of immunological graft injury in BKVN might be similar to that in AR (32). Fortunately, BK virus DNA tests in blood and urine are effective for differentiating BKVN from AR in the clinic. In addition, a more sensitive and specific method for BK virus detection including the presence of viral-specific miRNA has been introduced (33).

There are several limitations to this study. The quantification of mRNAs from urine samples is not easily undertaken because of the low purity of degraded mRNAs. There have always been issues of RNA normalization for quantitative PCR assay as well as RNA integrity, especially in urine samples (34). As with other studies using urine samples, limitations were connected to the fact that some of the urine samples from KTP patients had low integrity and could not be used. Moreover, the signature requires six-gene specific standard curves and is limited in clinical practice because it needs additional work. We designed the AR and STA groups in the training set and OGIs group as well as the AR and STA in the validation set. However, the AUC was reduced due to issues with validation, including the OGIs group. Furthermore, approximately 20% of urine samples did not pass quality control in clinical practice. Finally, the clinical utility of this urine gene signature in routine clinical practice, and could not be assessed in this sample collection cohort at the time of allograft biopsy. We validated our gene signature model in a large validation group that consisted of nearly all patients of the ARTKT sample collection study except for the training set. As shown in our ARTKT results, among patients undergoing renal biopsy after transplantation, the prevalence of rejection reaction was only 25.5%. These patients require additional diagnostic guidance in addition to the increased serum creatinine for allograft biopsy.

The positive predictive value of the urine gene signature was 63% and the negative predictive value was 80% in patients who had undergone allograft biopsy. Decision curve analysis suggests that the urine gene signature may complement or justify the allograft biopsy for diagnosing AR after kidney transplantation. However, the usefulness for clinical application of these gene sets and the selection of patients in need of clinical application should be evaluated through future multi-racial, prospective, and longitudinal studies.

In conclusion, this study performed a meta-analysis to discover biomarkers for the diagnosis of AR using cells in urine and validated the signature to diagnose and predict AR. Using ISH analysis, we showed that cells in urine could reflect ongoing kidney injuries or kidney allograft status. Therefore, our results demonstrate that the signature can be a noninvasive tool to assist with deciding whether to perform a biopsy in a recipient with a rise in creatinine and probably justifies a biopsy. However, it is necessary to further evaluate the performance of the signature for clinical usefulness in a prospective cohort.

## Data Availability Statement

All datasets generated for this study are included in the web resource: http://webtom.cabgrid.res.in/wbmstdb/.

## Ethics Statement

The studies involving human participants were reviewed and approved by Kyung Hee University Hospital at Gangdong. Written informed consent to participate in this study was provided by the participants’ legal guardian/next of kin.

## Author Contributions

J-WS performed qPCR, collected the data, statistical analyses of the results, and wrote the manuscript. SP carried out *in situ* hybridization experiments. KJ, C-DK, BC, JP, SJ, SL, JL, and YK collected the samples and participated in the acquisition of the clinical and pathology data of the enrolled patients. YHL and J-YM interpreted data analyses. DT and JS conducted bioinformatic analyses. S-HL participated in the conception, design of the study, and editing the manuscript. All authors contributed to the article and approved the submitted version.

## Funding

This work was supported by the Korean Health Technology R&D Project, Ministry of Health & Welfare, Republic of Korea (grant no. HI13C1232) and by the National Research Foundation of Korea Grant funded by the Korean government, Ministry of Science, and ICT (grant no. 2018M3A9E8078807).

## Conflict of Interest

The authors declare that the research was conducted in the absence of any commercial or financial relationships that could be construed as a potential conflict of interest.
