# Corrigendum: Non-Invasive Diagnosis for Acute Rejection Using Urinary mRNA Signature Reflecting Allograft Status in Kidney Transplantation

**DOI:** 10.3389/fimmu.2021.825243

**Published:** 2022-01-06

**Authors:** Jung-Woo Seo, Yu-Ho Lee, Dong Hyun Tae, Seon Hwa Park, Ju-Young Moon, Kyung Hwan Jeong, Chan-Duck Kim, Byung Ha Chung, Jae Berm Park, Yeong Hoon Kim, Junhee Seok, Sun Hyung Joo, Seung Hwan Lee, Jong Soo Lee, Sang-Ho Lee

**Affiliations:** ^1^ Core Research Laboratory, Medical Science Institute, Kyung Hee University Hospital at Gangdong, Seoul, South Korea; ^2^ Division of Nephrology, Department of Internal Medicine, Kyung Hee University Hospital at Gangdong, Seoul, South Korea; ^3^ School of Electrical Engineering, Korea University, Seoul, South Korea; ^4^ Division of Nephrology, Department of Internal Medicine, College of Medicine, Kyung Hee University, Seoul, South Korea; ^5^ Division of Nephrology, Department of Internal Medicine, Kyungpook National University School of Medicine, Daegu, South Korea; ^6^ Division of Nephrology, Department of Internal Medicine, Seoul St. Mary’s Hospital, College of Medicine, The Catholic University of Korea, Seoul, South Korea; ^7^ Department of Surgery, Sungkyunkwan University Samsung Hospital, Seoul, South Korea; ^8^ Division of Nephrology, Department of Internal Medicine, College of Medicine, Inje University Busan Paik Hospital, Busan, South Korea; ^9^ Department of Surgery, Kyung Hee University Hospital at Gangdong, Seoul, South Korea; ^10^ Division of Nephrology, Department of Internal Medicine, University of Ulsan College of Medicine, Ulsan, South Korea

**Keywords:** kidney, transplantation, non-invasive diagnosis, acute rejection, urinary mRNA

In the original article, we neglected to include “This work was supported by the Korean Health Technology R&D Project, Ministry of Health & Welfare, Republic of Korea (grant no. HI13C1232) and by the National Research Foundation of Korea Grant funded by the Korean government, Ministry of Science, and ICT (grant no. 2018M3A9E8078807)”.

In the original article, there was a mistake in the legend for **Figure 2A** as published. Incorrect word use in the legend changed the meaning of the statement. The correct legend appears below.

**Figure 2:**
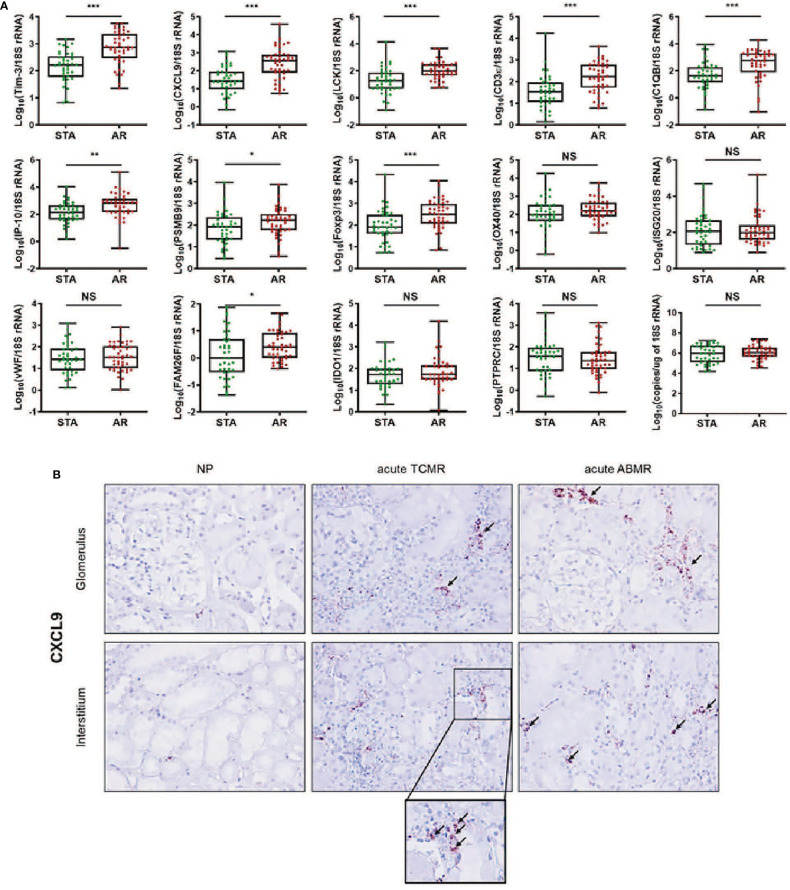
The expression levels of each mRNA between STA (n=45) and AR (n=58) were analyzed using absolute quantitative qPCR without pre-amplification. Each mRNA level was log10-transformed after each mRNA copy number was normalized with 18S rRNA copies (x10-6) in the QC-passed samples (STA, n=40; AR, n=44). **(A)** The levels of CXCL9, IP-10, C1QB, PSMB9, LCK, CD3ε, Foxp3, FAM26F, and Tim-3 mRNAs were significantly elevated in AR compared to STA, and for OX40, ISG20, vWF, IDO1, and PTPRC mRNAs, there was no difference. In the 18s rRNA used as an endogenous control, there was no difference between AR and STA. P values by the non-parametric Mann-Whitney test were expressed as the mean ± SE. NS: not significant, *P < 0.05, **P < 0.01 and ***P < 0.001 versus STA. Although LCK, Foxp3, and FAM26F mRNAs were statistically significant, these mRNAs were not detected in more than 10% of the QC-passed samples. Therefore, we excluded these mRNAs for further analysis. **(B)** CXCL9 mRNA expression in kidney biopsy tissues of NP, acute TCMR and acute ABMR groups was examined by ISH (original magnification x400). CXCL9 was distinctly expressed in the damaged tubules in kidney allografts of acute TCMR and predominantly in the peritubular capillary area in ABMR groups (black arrows). Scale bars: 50 μm.

In the published article, there was an error in affiliation 1. Instead of “Department of Core Research Laboratory, Medical Science Institute, Kyung Hee University Hospital at Gangdong, Seoul, South Korea”, it should be “Core Research Laboratory, Medical Science Institute, Kyung Hee University Hospital at Gangdong, Seoul, South Korea”.

The authors apologize for these errors and state that they do not change the scientific conclusions of the article in any way. The original article has been updated.

## Publisher’s Note

All claims expressed in this article are solely those of the authors and do not necessarily represent those of their affiliated organizations, or those of the publisher, the editors and the reviewers. Any product that may be evaluated in this article, or claim that may be made by its manufacturer, is not guaranteed or endorsed by the publisher.

