# Supplementary material for: Non-Invasive Diagnosis for Acute Rejection Using Urinary mRNA Signature Reflecting Allograft Status in Kidney Transplantation
Source: Front Immunol. 2021 Jun 10;12:656632. doi: 10.3389/fimmu.2021.656632 (PMC8222723; doi:10.3389/fimmu.2021.656632)
Supplement: Supplementary file 1 [file Table_1.docx]

**Supplemental Table 1. Public data sets for the meta-analysis**

| GEO dataset | Diagnosis of kidney transplant patients | | | Authors |
| --- | --- | --- | --- | --- |
|  | Stable graft function (STA) | Acute rejection (AR) | Total |  |
| GSE36059 | 281 | 130 | 411 | Halloran *et al.(1)* |
| GSE50058 | 58 | 43 | 101 | Khatri *et al.(2)* |
| GSE25902 | 96 | 24 | 120 | Naesens *et al.(3)* |
| GSE9489 | 7 | 15 | 22 | Raulf *et al.(4)* |
| Total | 442 | 212 | 654 |  |

**Supplemental Table 2. Top 10 candidate genes identified by the meta-analysis**

| Probeset ID | Gene symbol | Gene name | FDR | FC |
| --- | --- | --- | --- | --- |
| 203915_at | CXCL9 | C-X-X motif chemokine 9 | 5.22E-04 | 5.74 |
| 204533_at | IP-10 | C-X-C motif chemokine 10 | 2.02E-05 | 4.59 |
| 229390_at | FAM26F | Family with sequence similarity 26, member F | 1.00E-09 | 3.75 |
| 210029_at | IDO1 | Indoleamine 2,3-dioxygenase I | 1.22E-03 | 3.16 |
| 212588_at | PTPRC | Protein tyrosine phosphatase, receptor type C | 2.11E-04 | 2.95 |
| 202953_at | C1QB | Complement C1q B chain | 6.80E-04 | 2.83 |
| 204279_at | PSMB9 | Proteasome subunit beta 9 | 2.78E-04 | 2.68 |
| 204891_s_at | LCK | Lymphocyte cell-specific protein tyrosine kinase | 2.77E-03 | 2.15 |
| 204698_at | ISG20 | Interferon-stimulated exonuclease gene 20 | 1.00E-09 | 2.14 |
| 202112_at | VWF | von Willebrand factor | 3.00E-04 | 2.05 |

**Supplemental Table 3. The candidate genes through the literature review**

| Gene Symbol | Gene Name | Authors |
| --- | --- | --- |
| CD3E | CD3e molecule | Suthanthiran *et al*.(5) |
| Foxp3 | forkhead box P3 | Muthukumar *et al.(6)* |
| OX40 | Tumor necrosis factor receptor superfamily, member 4 (TNFRSF4), also known as CD134 | Afaneh *et al*.(7) |
| Tim-3 | T-cell immunoglobulin and mucin-domain containing-3 | Renesto *et al*.(8) |

**Supplemental Table 4. Total quantity and purity of RNA extracted from urine samples**

| RNA quantity | | | | |
| --- | --- | --- | --- | --- |
| Total RNA quantity (µg) | Total sample (N=494) | QC passed^†^ (N=402) | QC failed (N=92) | P value^‡^  QC passed vs QC failed |
| Median | 0.738 | 0.653 | 1.550 | < 0.05 |
| 25th percentile | 0.245 | 0.250 | 0.177 |  |
| 75th percentile | 1.680 | 1.643 | 1.881 |  |
| RNA Purity | | | | |
| RNA purity (A260/280 ratio) | Total sample (N=494) | QC passed ^†^ (N=402) | QC failed (N=92) | P value^‡^  QC passed vs QC failed |
| Median | 1.850 | 1.870 | 0.700 | < 0.001 |
| 25th percentile | 0.495 | 0.825 | 0.320 |  |
| 75th percentile | 2.005 | 2.020 | 1.980 |  |

The quantity (absorbance at 260 nm) and purity (ratio of the absorbance at 260 and 280 nm) of the RNA were measured using a NanoDrop® ND-2000 UV is spectrophotometer (Thermo Scientific).

† An RNA sample was considered to have passed QC if 18S rRNA and TGF-β1 mRNA levels were greater than or equal to 1x10^4^ and 1x10^2^ copies per microgram of total RNA, respectively.

‡ Two-tailed P value based on the Mann-Whitney test.

**Supplemental Table 5. 18S rRNA normalized, log-transformed levels of mRNA in training set (n=84)**

| Gene name | Stable (n=40) | Acute Rejection (n=44) | P Value^†^ | FDR* |
| --- | --- | --- | --- | --- |
| Tim-3 | 2.220 (1.773, 2.538) | 2.870 (2.475, 3.353) | <0.001 | <0.0001 |
| CXCL9 | 1.435 (0.988, 1.948) | 2.555 (1.885, 2.895) | <0.001 | 0.0001 |
| LCK | 1.280 (0.698, 1.870) | 1.975 (1.655, 2.488) | <0.001 | 0.0002 |
| CD3ε | 1.540 (1.063, 1.973) | 2.235 (1.725, 2.780) | <0.001 | 0.0002 |
| C1QB | 1.645 (1.170, 2.275) | 2.765 (1.898, 3.278) | < 0.001 | 0.0004 |
| FOXP3 | 1.915 (1.600, 2.478) | 2.505 (2.073, 2.978) | < 0.001 | 0.0011 |
| IP-10 | 2.145 (1.603, 2.648) | 2.810 (2.158, 3.143) | < 0.01 | 0.0035 |
| PSMB9 | 1.930 (1.328, 2.363) | 2.225 (1.760, 2.508) | < 0.05 | 0.0520 |
| FAM26F | -0.005 (-0.526, 0.694) | 0.402 (0.001, 0.925) | < 0.05 | 0.0477 |
| OX40 | 1.980 (1.623, 2.515) | 2.180 (1.883, 2.630) | 0.1677 | 0.2349 |
| IDO1 | 1.725 (1.275, 1.998) | 1.730 (1.493, 2.150) | 0.2181 | 0.2776 |
| ISG20 | 2.065 (1.323, 2.665) | 1.980 (1.608, 2.395) | 0.9002 | 0.9002 |
| PTPRC | 1.565 (0.876, 1.960) | 1.360 (0.982, 1.768) | 0.6414 | 0.6907 |
| vWF | 1.425 (0.919, 1.920) | 1.520 (1.020, 2.035) | 0.6003 | 0.6907 |
| 18S rRNA | 5.980 (5.125, 6.730) | 6.065 (5.665, 6.540) | 0.7068 | 0.7657 |

The levels of mRNA were absolutely measured without preamplification step by real-time quantitative PCR assays using gene specific oligonucleotide pairs and probes. If mRNA is undetected, the absolute value of the mRNA was calculated with standard curve by replacing 40 C_t_ value in the blank. Median value (lower, upper quartiles) of each mRNA measure normalized by 18s rRNA copy number (x10^-6^) and log_10_-transformed are shown. Data are shown for QC-passed samples.

† Two-tailed P value is based on the Mann-Whitney test.

*FDR (false discovery rate) is the result of multiple comparison correction.

**Supplemental Table 6. ROC curve analysis of individual mRNA to differentiate AR from STA in training set**

| Type of mRNA | Area Under the Curve | 95% Confidence Interval | | | P value |
| --- | --- | --- | --- | --- | --- |
| Tim-3 | 0.81 | 0.711 | 0.899 | | < 0.001 |
| CXCL9 | 0.78 | 0.685 | 0.881 | | < 0.001 |
| LCK | 0.76 | 0.660 | 0.867 | | < 0.001 |
| CD3ε | 0.76 | 0.653 | 0.860 | | < 0.001 |
| C1QB | 0.74 | 0.629 | 0.853 | | < 0.001 |
| FOXP3 | 0.72 | 0.613 | 0.832 | | < 0.001 |
| IP-10 | 0.70 | 0.586 | 0.811 | | < 0.01 |
| PSMB9 | 0.64 | 0.515 | 0.755 | | < 0.05 |
| FAM26F | 0.64 | 0.516 | 0.763 | | < 0.05 |
| OX40 | 0.59 | 0.463 | 0.712 | | 0.168 |
| IDO1 | 0.58 | 0.454 | 0.702 | | 0.218 |
| ISG20 | 0.51 | 0.380 | 0.636 | | 0.900 |
| PTPRC | 0.47 | 0.343 | | 0.598 | 0.641 |
| vWF | 0.53 | 0.408 | | 0.659 | 0.600 |
| 18S rRNA | 0.52 | 0.395 | | 0.653 | 0.707 |

Receiver-operating-characteristic (ROC) curve analyses of the urinary mRNA measures to differentiate AR from STA are shown. The AUC value of Tim-3 mRNA was best to differentiate the two groups.

**Supplemental Table 7. 18S rRNA normalized, log-transformed levels of mRNA in validation set (n=318)**

| Gene name | Stable (STA, n=122) | Acute Rejection (AR, n=57) | Borderline Changes (BC, n=42) | BK Virus Nephropathy (BKVN, n=12) | Other Graft Injuries  (OGI, n=85) | P Value^†^ | Compared between groups | P Value^‡^ STA Vs. | P Value^‡^ AR Vs. |
| --- | --- | --- | --- | --- | --- | --- | --- | --- | --- |
| Tim-3 | 1.470 (1.055, 1.925) | 2.410 (2.245, 2.810) | 1.680 (1.390, 2.173) | 2.335 (2.110, 2.895) | 2.040 (1.485, 2.420) | <0.001 | AR BC BKVN OGI | <0.001 <0.05 <0.001 <0.001 | <0.001 0.728 <0.001 |
| CXCL9 | 0.750 (0.022, 1.610) | 2.120 (1.390, 2.430) | 1.495 (0.713, 1.833) | 2.640 (2.150, 3.175) | 1.440 (0.696, 1.905) | <0.001 | AR BC BKVN OGI | <0.001 <0.01 <0.001 <0.01 | <0.001 <0.01 <0.001 |
| CD3ε | 1.380 (0.677, 1.853) | 2.170 (1.790, 2.535) | 1.820 (1.558, 2.330) | 1.965 (1.748, 2.385) | 1.680 (1.075, 2.130) | <0.001 | AR BC BKVN OGI | <0.001 <0.001 <0.01 <0.05 | <0.05 0.375 <0.001 |
| C1QB | 1.625 (0.898, 2.078) | 2.450 (2.120, 2.850) | 1.960 (1.423, 2.263) | 2.525 (2.285, 3.050) | 2.150 (1.475, 2.725) | <0.001 | AR BC BKVN OGI | <0.001 <0.05 <0.001 <0.001 | <0.001 0.232 <0.01 |
| IP-10 | 1.445 (0.993, 2.073) | 2.480 (1.930, 2.765) | 1.775 (1.315, 2.235) | 3.060 (2.535, 3.188) | 1.770 (1.200, 2.300) | <0.001 | AR BC BKVN OGI | <0.001 0.103 <0.001 0.085 | <0.001 <0.01 <0.001 |
| PSMB9 | 0.959 (0.442, 1.625) | 1.880 (1.625, 2.215) | 1.120 (0.749, 1.578) | 1.645 (1.320, 2.185) | 1.460 (1.003, 1.940) | <0.001 | AR BC BKVN OGI | <0.001 0.545 <0.01 <0.001 | <0.001 0.429 <0.001 |

The levels of mRNA were absolutely measured without preamplification step by real-time quantitative PCR assays using gene specific oligonucleotide pairs and probes. Median value (lower, upper quartiles) of each mRNA measure normalized by 18s rRNA copy number (x10^-6^) and log_10_-transformed are shown. Data are shown for QC-passed samples.

† P value is based on the Kruskal-Wallis test, and ‡ P value is based on the Mann-Whitney test between two groups.

**Supplemental Figure 1. Validation of six significant mRNAs in independent samples**

The absolute quantitative qPCR was performed to validate six significant mRNAs in 391 independent samples, which consist of STA (n=153), AR (n=68), BC (n=58) for TCMR, BKVN (n=15), and OGIs (n=97) including ATN (n=30), CNI (n=28) toxicity, GN (n=27), and IF/TA (n=12). Each mRNA level was log_10_-transformed after each mRNA copy number was normalized with 18S rRNA copies (x10^-6^) in the QC-passed samples. The levels of CXCL9, IP-10, C1QB, PSMB9, CD3ε, and Tim-3 mRNAs were significantly elevated in AR compared to STA, BC, and OGIs. CXCL9 and IP-10 mRNA levels in BKVN were significantly higher than in AR. P values using pairwise comparisons by the non-parametric Mann-Whitney test were expressed as the mean±SE. * P<0.05, ** P<0.01, and *** P<0.001 versus STA; ^#^ P<0.05, ^##^ P<0.01, and ^###^ P<0.001 versus AR.

**
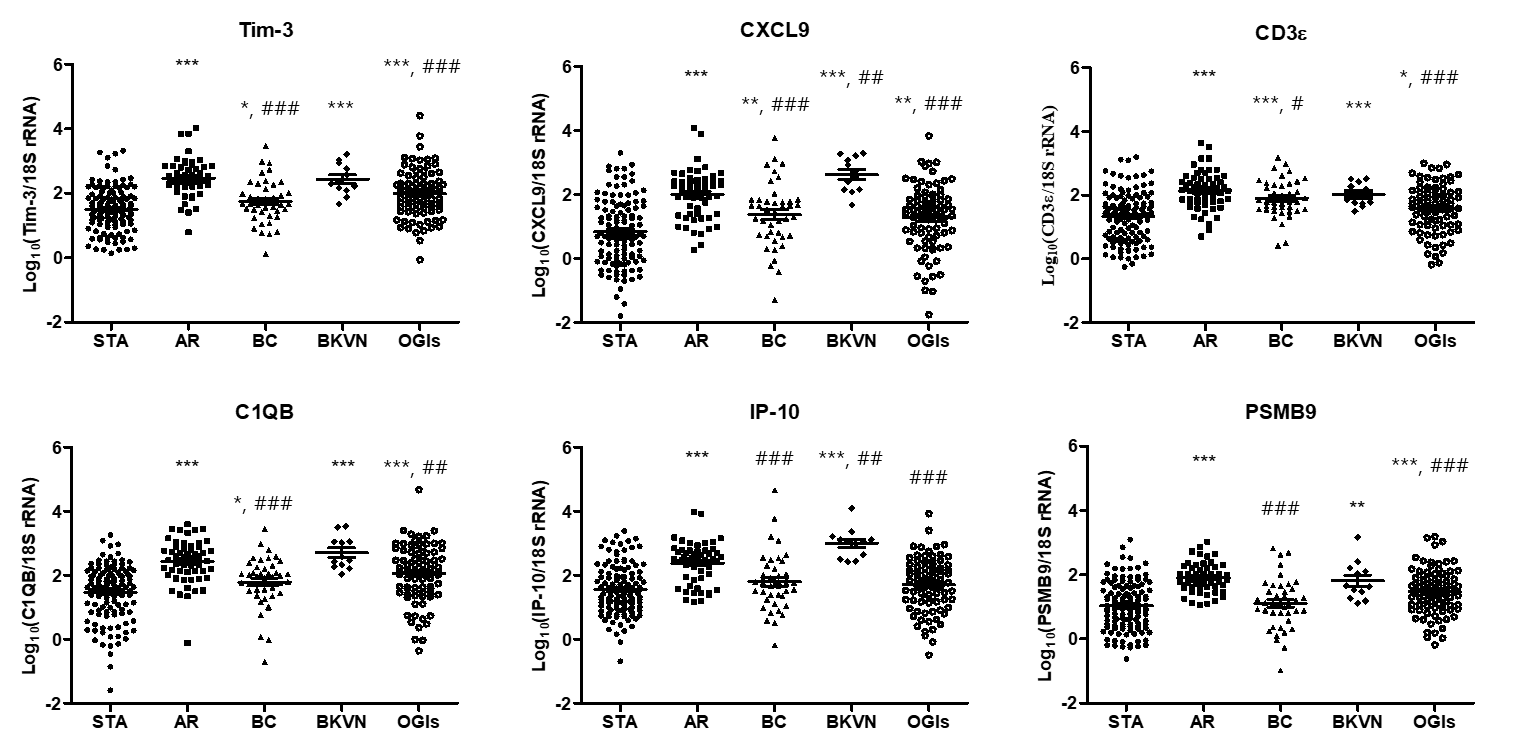
**

**Supplemental Figure 2.** **Gene set analysis of significant genes identified by meta-analysis**

Gene set enrichment analysis of top 100 significant genes identified by meta-analysis. Among 10 candidates identified by meta-analysis, 9 genes were associated to inflammatory response and immune system.

**
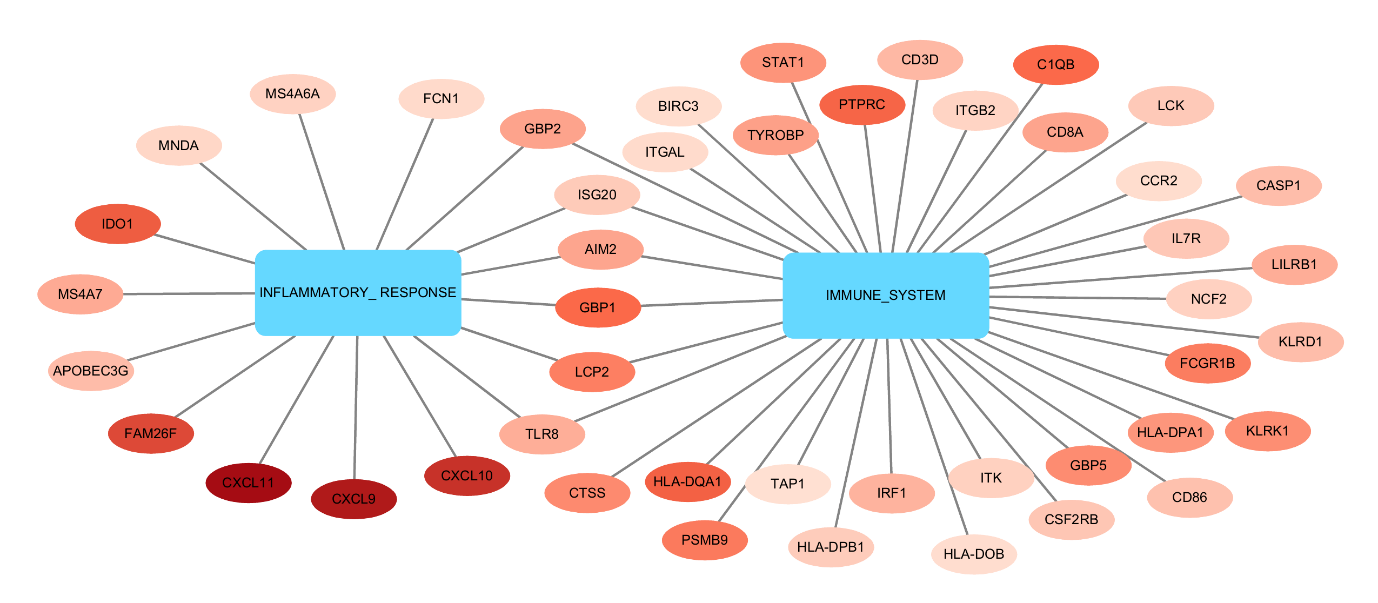
**

**References**

1. J. Reeve, J. Sellares, M. Mengel, B. Sis, A. Skene, L. Hidalgo, et al.: Molecular diagnosis of T cell-mediated rejection in human kidney transplant biopsies. *Am J Transplant*, 13(3), 645-55 (2013) doi:10.1111/ajt.12079

2. P. Khatri, S. Roedder, N. Kimura, K. De Vusser, A. A. Morgan, Y. Gong, et al.: A common rejection module (CRM) for acute rejection across multiple organs identifies novel therapeutics for organ transplantation. *J Exp Med*, 210(11), 2205-21 (2013) doi:10.1084/jem.20122709

3. M. Naesens, P. Khatri, L. Li, T. K. Sigdel, M. J. Vitalone, R. Chen, et al.: Progressive histological damage in renal allografts is associated with expression of innate and adaptive immunity genes. *Kidney Int*, 80(12), 1364-76 (2011) doi:10.1038/ki.2011.245

4. P. Saint-Mezard, C. C. Berthier, H. Zhang, A. Hertig, S. Kaiser, M. Schumacher, et al.: Analysis of independent microarray datasets of renal biopsies identifies a robust transcript signature of acute allograft rejection. *Transpl Int*, 22(3), 293-302 (2009) doi:10.1111/j.1432-2277.2008.00790.x

5. M. Suthanthiran, J. E. Schwartz, R. Ding, M. Abecassis, D. Dadhania, B. Samstein, et al.: Urinary-cell mRNA profile and acute cellular rejection in kidney allografts. *N Engl J Med*, 369(1), 20-31 (2013) doi:10.1056/NEJMoa1215555

6. T. Muthukumar, D. Dadhania, R. Ding, C. Snopkowski, R. Naqvi, J. B. Lee, et al.: Messenger RNA for FOXP3 in the urine of renal-allograft recipients. *N Engl J Med*, 353(22), 2342-51 (2005) doi:10.1056/NEJMoa051907

7. C. Afaneh, T. Muthukumar, M. Lubetzky, R. Ding, C. Snopkowski, V. K. Sharma, et al.: Urinary cell levels of mRNA for OX40, OX40L, PD-1, PD-L1, or PD-L2 and acute rejection of human renal allografts. *Transplantation*, 90(12), 1381-7 (2010) doi:10.1097/TP.0b013e3181ffbadd

8. P. G. Renesto, V. C. Ponciano, M. A. Cenedeze, N. O. Saraiva Camara and A. Pacheco-Silva: High expression of Tim-3 mRNA in urinary cells from kidney transplant recipients with acute rejection. *Am J Transplant*, 7(6), 1661-5 (2007) doi:10.1111/j.1600-6143.2007.01795.x
